# Do inexperienced nurses in the lactation period experience workplace violence? A qualitative study

**DOI:** 10.3389/fpubh.2024.1387976

**Published:** 2024-06-25

**Authors:** Runpeng Chen, Ruiwen Wang, Dongyang Wang, Qinghua Wang, Xinghui Liu

**Affiliations:** ^1^Department of Nursing, Binzhou Medical University, Yantai, China; ^2^Department of Anesthesiology, Yantai Yuhuangding Hospital, Qingdao University, Yantai, China; ^3^Department of Nursing, The Third People’s Hospital of Henan Province, Zhengzhou, China; ^4^Shandong Vheng Data Technology Co., Ltd., Yantai, China

**Keywords:** inexperienced nurses, breast feeding period, workplace violence, challenges, role confusion

## Abstract

**Introduction:**

Among clinical healthcare personnel, nurses face the highest proportion of workplace violence, which has a significant impact on their physical and mental well-being as well as their personal and professional lives. However, little is known about the effects of workplace violence on inexperienced breastfeeding nurses and their experiences during and after breastfeeding when they return to work. This study aimed to explore the experiences of inexperienced breastfeeding nurses who encountered workplace violence and its resulting impacts.

**Methods:**

This study employed a descriptive qualitative design. Semi-structured in-depth interviews were conducted with 20 nurses working in various positions and departments at three tertiary hospitals. Purposive and maximum variation sampling techniques were employed. The interview data were analyzed using Colaizzi’s method, and the research findings were reported according to Consolidated Criteria for Reporting Qualitative Studies (COREQ)standards.

**Results:**

Inferences regarding workplace violence and risks for inexperienced breastfeeding nurses included physical labor (such as lifting heavy objects and performing cardiopulmonary resuscitation), conflicts, inadequate job skills, role confusion, occupational exposure risks, patient violence, and pressure from older adults. An inductive thematic investigation revealed the “Challenges faced during breastfeeding,” “Conflicting professional and family roles,” “Out of balance,” and “Coping strategies.”

**Conclusion:**

Inexperienced breastfeeding nurses experience several negative consequences due to workplace violence. Therefore, it is essential to plan and implement preventive strategies and management programs that specifically target workplace violence among inexperienced breastfeeding nurses.

## 1 Introduction

The World Health Organization defines Workplace Violence (WPV) as “incidents in which employees are abused, threatened, or assaulted in circumstances related to their work, involving explicit or implicit challenges to their safety, well-being, or health” (1). Research has shown that healthcare workers, particularly those in medical institutions, are most affected by WPV, possibly five times more than workers in other sectors (2). WPV against healthcare workers is recognized as a global public health safety issue; among healthcare workers, nurses have the highest frequency of patient contact, thus experiencing a higher proportion of WPV incidents (3). A study revealed that WPV will result in nine million nurses leaving their profession globally in 2022, leading to a global shortage of nursing personnel and causing significant losses to the healthcare sector (4).

Forms of violence against nurses primarily include verbal, physical, psychological, and sexual violence. Among these, verbal violence has the highest proportion, followed by psychological violence, while physical violence accounts for a smaller proportion; sexual violence is the least tolerated by nurses (5, 6). In recent years, the incidence of cyberbullying targeting healthcare professionals has been increasing, including the malicious interception of audio and video recordings in medical settings and the sharing of unethical videos, audio, and explicit content (7). The WPV legislation varies across countries, resulting in different global prevalence rates. A systematic review and meta-analysis focusing on healthcare professionals reported the highest prevalence of WPV in Australia, followed by North American countries and Asia (8). In recent years, there has been a declining trend in WPV prevalence in Asian countries, while Europe has the lowest prevalence but has shown an increasing trend in recent years (8). Among healthcare professionals, 61.9% have experienced WPV, 42.5% have experienced non-physical violence (verbal violence, threats, sexual harassment), and 24.4% have experienced physical violence in the past year (8). A cross-sectional study in China reported that the incidence rates of physical and psychological violence against nurses were 9.6 and 59.64%, respectively (9). Zhang et al. found that 65.2% of nurses had experienced verbal violence and 5.9% had experienced sexual violence (10). Risk factors for nurses’ experience with WPV include age, work experience, working hours, shift work, work schedules, full-time employment status, presence of WPV reporting procedures, patient types, direct physical contact with patients, and presence of reporting systems (11, 12). Among these factors, inexperienced nurses are characterized by being under the age of 30, having a short work tenure, and lacking sufficient work experience, which are significant risk factors for WPV (13). Inexperienced nurses refer to those with a clinical nursing work tenure of ≤5 years (14). Studies have suggested that younger nurses with lower qualifications have an increased risk of experiencing WPV. They reported a higher frequency of physical violence, longer shifts, and more night shifts (15). Breastfeeding is another important characteristic of female nurses in this age group. Research indicates that approximately 50% of the nurses in the United States are of reproductive age and commonly experience the challenges of returning to work and balancing their personal lives (16).

In China, the number of inexperienced nurses during the breastfeeding period is substantial (17). After obtaining a bachelor’s degree in nursing, most nursing students enter clinical nursing positions, and many of them get married and have children within a short period. The maternity leave policy in China stipulates that women who give birth should be entitled to 98 days of paid maternity leave (18). Afterward, they return to their work positions and are allowed 1 h of breastfeeding time during each working day until planned weaning (18). For inexperienced nurses, combining breastfeeding with work responsibilities during the breastfeeding period could be exhausting. Furthermore, working full-time poses significant challenges to exclusive breastfeeding for 6 months (19). In addition to the conflict between work and breastfeeding, experiencing WPV makes their situation even more difficult and can lead to various social, familial, and psychological problems. Currently, pediatric, emergency, and psychiatric departments have the highest WPV rates (3). Scholars have conducted qualitative research on the WPV experienced by nurses in pediatric emergency departments to explore the types, causes, response methods, and personal and professional consequences of violence (20). One study focusing on psychiatric nurses conducted a systematic review of the occurrence rate, influencing factors, and consequences of WPV, providing recommendations for the development of standardized tools and preventive strategies to address this issue (21). Regarding the challenges faced by nurses returning to work after maternity leave, one study recruited nurses who had recently experienced parental leave from an emergency department in a hospital in the Midwest United States. The research described their experiences and the balance between their personal lives and work, but primarily focused on women’s rights, breastfeeding, and infant care issues, without addressing WPV (16). Although several qualitative studies have explored nurses’ encounters with WPV, to our knowledge, none has specifically investigated the experiences and responses of inexperienced nurses who are breastfeeding. Given the varying likelihood and severity of WPV across countries, along with differences in policies regarding breastfeeding among female employees, it follows that inexperienced nurses who are also breastfeeding will exhibit a range of inherently distinctive experiences and reactions (4). Therefore, the objective of this study was to offer a detailed portrayal and narrative of the experiences and subsequent impact on inexperienced nurses during the breastfeeding period following their exposure to WPV.

## 2 Methods

### 2.1 Design

This study adopted a qualitative descriptive design based on a phenomenological approach, using face-to-face semi-structured interviews to collect data from participants. The qualitative descriptive design was interpretive, descriptive, and highly contextual, following the phenomenological research principle that “derived realities are composed of events perceived by those who experience them” (22). Phenomenological methods, an important approach in qualitative research, focus on the description of the subject’s awareness of the experience, emphasizing the characteristics of thinking that exist when the subject is experiencing a phenomenon, with a logical relationship between the phenomenon described and the category of description (23). Semi-structured interviews, the main form of interviews in qualitative research, need to be agreed upon with the participants in advance of the time and place. The outline of the interview began with some open-ended questions, through the conversation, the collection of data, and the participant’s description of the life experience and event information to dig out the new research points, in the interview based on the flexibility of the outline of the interview at the right time to ask new interview questions, until you get all the event information data. Afterward, the researcher then distilled the terminology from the data consisting of the participants’ descriptions, showing all the descriptive categories and providing a comprehensive summary of the research event (24, 25).

In this study, the interviewers conducted structured questioning of breastfeeding nurses who had experienced WPV to gather information on the experiences and feelings of the participants. Through summarization, the data obtained had both commonalities and characteristics. Initially, some representative sub-thematic terms were summarized; thereafter, all the sub-thematic terms were summarized and categorized to come up with the most descriptive ones to make all the results logically coherent.

### 2.2 Participants

This study was supported by three tertiary hospitals in Shandong Province, China. These hospitals provide relevant information to the nursing personnel. Some workplaces may have additional maternity leave provisions, and the participants of this study took 158 days of maternity leave. Participants were recruited using both purposive and maximum variation sampling methods, which were subjective and selective, allowing the researcher to decide on the inclusion of participants based on a variety of criteria such as professional background on the research question and the ability and willingness of the participant. Maximum variation sampling, which provides a deeper understanding of a phenomenon based on a wide range of perspectives of interest to the researcher, ranges from typical to individual phenomena; both sampling methods can help researchers better generalize common themes in the sample (26). All participants volunteered and were assured of their anonymity. The researchers contacted the participants, explained the research purpose and procedures, and obtained informed consent. Twenty clinical, low-experience lactating nurses were interviewed, representing various departments, including outpatient clinics, intensive care units, emergency departments, pediatric wards, internal medicine and surgery wards, and operating rooms, from the three participating hospitals. The inclusion criteria were as follows: currently employed registered nurses, lactating mothers, experienced WPV, less than or equal to 5 years of work experience (14), and willingness to participate with informed consent. Table 1 provides a brief description of participants’ demographic characteristics.

**Table 1:** Participants’ demographic characteristics (*N* = 20).

Participant number	Age (years)	Years of work experience (years)	Educational background	Working department	Duration of breastfeeding (months)
N1	26	3	Bachelor’s degree	Department of cerebral surgery	6 months
N2	26	3	Bachelor’s degree	Emergency room	6 months
N3	26	2	Bachelor’s degree	Department of cardiac surgery	7 months
N4	25	2	Bachelor’s degree	Department of gynecology	9 months
N5	26	3	Bachelor’s degree	Department of gastrointestinal surgery	8 months
N6	27	1	Master’s degree	Department of cardiac surgery	8 months
N7	24	1	Bachelor’s degree	Department of pediatrics	6 months
N8	26	2	Bachelor’s degree	Emergency room	7 months
N9	25	3	Bachelor’s degree	Respiratory medicine	8 months
N10	23	1	Bachelor’s degree	Department of infectious diseases	6 months
N11	22	1	Bachelor’s degree	Department of gastroenterology	6 months
N12	27	2	Master’s degree	Department of neurology	9 months
N13	23	1	Bachelor’s degree	Operating room	6 months
N14	24	2	Bachelor’s degree	Department of gastroenterology	9 months
N15	26	1	Master’s degree	Department of urology	6 months
N16	24	2	Bachelor’s degree	E.N.T. department	11 months
N17	23	1	Bachelor’s degree	Department of obstetrics	9 months
N18	25	3	Bachelor’s degree	Operating room	7 months
N19	24	1	Bachelor’s degree	Department of ophthalmology	8 months
N20	23	1	Bachelor’s degree	Department of traumatology	7 months

### 2.3 Data collection

The data collection period for this study was from September to December 2023. The research team conducted face-to-face semi-structured interviews with each participant. The participants chose interview locations, and the starting time was determined based on their preferences, with the flexibility to end the interviews as needed.

The research team consisted of four members, all of whom were professional registered nurses with experience in qualitative research and relevant certifications. To refine the interview guide in terms of format and content, three pilot interviews were conducted with participants. Subsequently, the interview guide was modified based on the interview format and content, resulting in the final version presented in Table 2. Throughout the interviews, new questions were raised flexibly and promptly, according to specific circumstances. All interviews were audio recorded using a semi-structured interview guide. A total of 35 interviews were conducted without time limitations until data saturation was achieved. Within 24 h, the audio recordings were transcribed into written text. All the participants provided written informed consent (Table 2).

**Table 2:** Interview outline.

Interview outline
1.Has your work and life changed since you started breastfeeding? If so, what were the specific changes?
2.How do you reflect upon your current lifestyle and work situation? (How would you describe your current lifestyle and work status?)
3.Could you please provide specific instances of WPV that you have encountered during the breast feeding period? Could you elaborate on those incidents?
4.How did you respond when faced with WPV during the breast feeding period?
5.What kind of physical and psychological changes did you observe in yourself after experiencing WPV during the breast feeding period?
6.What strategies or interventions do you believe can help reduce the impact of WPV on individuals in the breast feeding period? This could include actions from hospitals, families, or self-initiated measures.

### 2.4 Data analysis

During the data analysis process, the researchers utilized the method of content analysis to systematically categorize and summarize the interview data, following the guidelines of “Consolidated Criteria for Reporting Qualitative Research” (27) and the strategies for ensuring trustworthiness (28).

The researchers conducted data analysis using the Colaizzi method (29) and managed the data using Nvivo12 software. Each answer provided by the participants in response to the interview guide was meticulously read and analyzed repeatedly. The analysis process initially focused on comprehending semantic aspects. Subsequently, each researcher independently coded the themes emerging during the analysis. Finally, the entire research team integrated and analyzed all potential themes and codes, resulting in the final thematic coding and category information. A shared thematic vocabulary program and spreadsheet were used for consolidation. The data analysis process continued until themes, subthemes, and data saturation were established from the interview information. The steps involved in the data analysis and processing are presented in Table 3.

**Table 3:** Data analysis and processing steps.

Data analysis and processing steps	
1	Researchers engage in iterative listening to audio recordings and in-depth reading of transcribed interview content, meticulously immersing themselves in each interview to ensure thorough familiarization.
2	Systematically code the distinctive information derived from analyzing the interview data, establishing a coherent connection between the raw interview data and research objectives, and meticulously organize all the codes in a structured manner.
3	Categorize the established codes into latent themes and subsequently identify and define sub-themes that are pertinent to each latent theme, capturing the nuanced intricacies of the data.
4	Construct a comprehensive visual representation, such as an illustrative diagram, incorporating the encoded themes and their corresponding sub-themes. Engage in further examination of the interview data, employing necessary refinements, additions, or substitutions to accurately reflect the underlying themes and sub-themes.
5	Conduct a rigorous analysis of the final illustrative diagram to ascertain precise definitions and aptly label each theme, ensuring conceptual clarity and coherence.
6	Synthesize the research findings based on the final themes and their corresponding sub-themes, eloquently presenting the rich qualitative insights and interpretations that emerged from the study.

### 2.5 Trustworthiness

Strategies for ensuring trustworthiness (28): (1) Credibility: The researchers meticulously analyzed and synthesized the interview notes and audio recordings obtained during the interview process. The identified themes were carefully verified and compared with the participants’ perspectives to ensure that the collected data accurately reflected their experiences and thoughts. This rigorous process helped to establish the credibility of the research findings. (2) Transferability: Purposeful sampling methods are employed to provide a comprehensive description of the research phenomenon. These methods allowed a detailed exploration of the data collection and analysis processes, facilitating the transferability of the findings to other contexts. (3) Dependability: The researchers ensured dependability of the study through triangulation. External experts were involved in the review, evaluation, and comparison of the research findings to enhance the trustworthiness and dependability of the results. (4) Confirmability: To mitigate researcher bias, researchers acknowledged the limitations and potential influences of their research approaches. They provided in-depth methodological descriptions and utilized visual aids such as charts to document the review process and steps taken. These measures ensure the confirmation and integrity of the research findings.

### 2.6 Ethics considerations

This study was approved by our research institution (2023-004). The study adhered to the ethical standards outlined in the Declaration of Helsinki, ensuring that all participants voluntarily participated and signed informed consent forms. They were informed about the purpose of the study, and appropriate measures were taken to ensure anonymity and protect their privacy. There were no conflicts of interest between the participants and the researchers.

## 3 Results

During the data analysis process, a total of four hundred and thirty initial codes were generated, which were subsequently grouped into twelve subthemes. Through careful analysis, four main themes emerged from the data: “challenges faced during the breastfeeding period,” “warranting professional and family roles,” “out of balance,” and “coping strategies” (Table 4).

**Table 4:** Themes and subthemes.

Theme	Subtheme
Challenges faced during the breast feeding period	Health effects
	Lack of sleep
	Conflict between Work and Breastfeeding
	Harassment Fear
Conflicting Professional and Family Roles	Job-Ability Mismatch
	Role Confusion
Out of balance	Workplace conflicts
	Occupational Exposure Panic
	Relative pressure
Coping strategies	Self-adjustment
	Effective Communication
	Facing up to the dilemma

### 3.1 Theme 1: challenges faced during the breastfeeding period

#### 3.1.1 Health effects

Several participants reported experiencing breast pain and discomfort during nursing procedures such as cardiopulmonary resuscitation, lifting heavy objects, and performing arm and hand movements. This pain often results in a slowdown of work processes. Some participants faced criticism and discipline from their superiors, who perceived their reduced efficiency as a deliberate lack of commitment. Participants were hesitant to discuss the physiological challenges they encountered during breastfeeding. Consequently, they experience feelings of frustration, injustice, and an increased mental burden. Ongoing breast pain leads to symptoms such as elevated body temperature, headaches, palpable breast lumps, tenderness, and cessation of breastfeeding. In severe cases, the participants had to request leave to seek treatment, which significantly affected their health and work status.

“I could not even lift my arm. On that day, during patient resuscitation, my breasts were too painful, possibly indicating inflammation. I could not perform Cardio Pulmonary Resuscitation properly and I could not be assisted. The nurse in charge perceived this as me avoiding work and punishing me. I truly felt distressed” (N2).

“There are instances when changing fluids for patients becomes extremely painful, as it involves my breasts. I must endure pain without showing it to the patient. Additionally, I feel that some colleagues do not understand my situation, as they may not have encountered these problems during their breastfeeding experiences. However, the pain that I endured was genuine. I have breast lumps and a persistent fever, and the only option left is to take leave” (N9).

#### 3.1.2 Lack of sleep

Some participants reported that busy daytime work and physical exhaustion combined with the need to breastfeed their children multiple times at night resulted in inadequate sleep. This lack of sleep leads to poor mental well-being. The nurse in charge perceived their work attitude as inappropriate and publicly criticized them harshly during morning meetings, causing significant anxiety and guilt, and affecting their emotional state.

“I do not get enough rest at night, and I feel like my energy cannot keep up. The department is too busy, and I feel guilty in front of the patients. Today, the nurse-in-charge said that I was not taking my work seriously and scolded me for a long time. I really wish that she could understand my situation. Does not she experience similar difficulties during her breastfeeding period?” (N8, sighing).

“I wake up many times during the night to breastfeed my baby and change diapers, these things cause me to be very sleepy during the day, my coworkers know that I am in the middle of breastfeeding and are very sympathetic, and the nurse practitioner is aware of my situation, but she has approached me many times to talk to me because of my poor mental state” (N18).

#### 3.1.3 Conflict between work and breastfeeding

The majority of participants expressed that their workload in the department was substantial. Although their breastfeeding shifts were delayed by only 1 hour, their individual workloads remained unchanged. If they could not complete their tasks on time, the nurse manager exerted continuous pressure on them, while colleagues perceived them as having low work efficiency and inadequate abilities. Consequently, they frequently worked overtime and were unable to return home on time. At times, they had no choice but to have their husbands bring their children to the department in order to facilitate timely breastfeeding. This situation has led to discontent among husbands. Some husbands believed that delayed breastfeeding was due to their wives’ poor work efficiency and suggested early weaning and the use of formula milk to alleviate the challenges posed by the conflicting demands of breastfeeding and work. In extreme cases, husbands attributed their wives’ professional incompetence to their children’s crying, resulting in conflict between spouses.

“My workload is overwhelming, making it impossible to leave on time. By the time I get home, my child is starving. My husband brings the child to the breastfeeding department, which is when the complaints begin. We end up arguing. I genuinely do not want them to come, but I fear my child going hungry” (N6).

“Due to my excessive workload, my family constantly worries about insufficient milk supply and keeps making negative remarks” (N7).

“My husband suggested early weaning for convenience, but I am reluctant. I am concerned about the well-being of my child” (N5).

#### 3.1.4 Harassment fear

A minority of participants reported that breastfeeding caused physiological breast enlargement, which in turn led to harassment from male caregivers. Most nurses found this experience shameful and chose to endure it silently, attempting to forget the incident. However, a few nurses resisted the harassment and actively advocated for their rights. Those who encountered such situations expressed fear of encountering male patients or caregivers in their future work, dreading the possibility of facing harassment. Additionally, they experienced feelings of shame, believing that the harassment was the result of their physiological state.

“In the emergency department, intoxicated male patients and caregivers often continuously stare at their breasts. Some used offensive language and engaged in physical advances. I experience such harassment twice a month, which is truly bothersome. Initially, I tried to tolerate it, but eventually, I could no longer bear it. There is another nurse who is breastfeeding like me and she has encountered similar incidents. When I asked her why she did not resist, she replied, ‘Let it go, sigh!’” (N8).

“Breastfeeding has caused my breasts to swell a bit more than they were, and sometimes, when nursing male patients, their eyes linger on my breasts, and these stares make me uncomfortable!” (N13).

### 3.2 Theme 2: conflicting professional and family roles

#### 3.2.1 Job-ability mismatch

All participants reported that the hospital’s maternity leave policy allowed for a five-month period of breastfeeding at home before returning to work. However, nurses often struggle with an increased sense of discomfort owing to the rapid transition between their professional and family roles. Their limited work experience and insufficient skills and abilities contribute to difficulties comprehending patient conditions, unfamiliarity with medical equipment usage and nursing procedures, inadequate communication skills, and the inability to effectively manage nursing tasks. Consequently, work efficiency is compromised, leading to a lack of confidence in interacting with patients. Some patients question nurses’ competencies, perceive obstacles in nursing procedures, or deliberately conceal nursing information. This skepticism extends to severe doubts about the overall quality of hospital staff and medical services, often expressed through verbal abuse, and in extreme cases, physical violence towards nurses.

“Once I start my shift, I feel completely overwhelmed. Although I received some preemployment training when I first joined, I became pregnant shortly thereafter, which limited my opportunities to gain substantial work experience. Now I feel ill and equipped to handle my job responsibilities. The department was equipped with various medical devices, many of which I’m not proficient in using. This lack of proficiency results in low work efficiency and patients begin to doubt their nursing skills. The pressure and exhaustion were overwhelming. I strongly believe that receiving specialized technical training for my position would greatly benefit me” (N15).

“I have just been at work for a few days and I have been really stressed these past few days, feeling very out of sorts… I feel like my pregnancy has made my memory worse, some of my nursing operations are not particularly skilled, I cannot keep up with the pace of the department, and I almost got a complaint from a patient today” (N4).

#### 3.2.2 Role confusion

Some participants mentioned that after breastfeeding their children at home, they questioned them about their absence when they arrived at work (even if there were no nursing issues to address). Even after providing explanations, some patients maliciously complained about the nurses’ perceived irresponsibility. Family-related issues suffocated them. After their children were born, their husbands chose to remain passive in caring for certain matters, citing fear of making mistakes. Long-term frustration with work and family life led nurses to experience role confusion. They were unsure about how to balance their work, family, and child-rearing responsibilities.

“Some patients would ask me where I had been after I arrived for my shift. Even after the explanation, some patients did not understand, and instead started complaining about the hospital’s staffing issues, intentionally lodging complaints against me” (N20).

“I cannot be in two places at once, I cannot divide my attention. I want to provide timely care to my patients, but I cannot let go of my children. My husband does not want to take any responsibility” (N5, sobbing).

### 3.3 Theme 3: out of balance

#### 3.3.1 Workplace conflicts

Some participants reported that nurses in the breastfeeding period were entitled to 1 hour of breastfeeding time per day, during which other day-shift nurses temporarily took care of their shared patients. However, this has resulted in dissatisfaction among certain nurses, leading to targeted hostility, baseless accusations, and passive-aggressive behavior. These nurses viewed their breastfeeding breaks as legitimate entitlements and expressed their unwillingness to yield to or proactively mend relationships with colleagues. Consequently, some nurses reached the point of detachment from their coworkers, creating an uncomfortable working environment.

“Some colleagues believe that I am doing less work, while they have to do more, which causes them to treat me indifferently and display sour expressions. Why should I initiate a conversation with them? My breastfeeding breaks are within my rights” (N3).

“I just had a bit of a disagreement with a colleague on my unit today, I arrived ten minutes late today on top of my one-hour nursing time because of some traffic on the road, but she complained to the head nurse that I do not take my job seriously, I went to her and theorized about it, and I was really especially angry!” (N17).

#### 3.3.2 Occupational exposure panic

Some participants indicated that they worked in departments that admitted patients with infectious diseases, thus posing occupational exposure risks. Consequently, they are deeply concerned about the potential transmission of contagious illnesses to their families, particularly concerning their children’s health. This high level of anxiety places them under significant mental strain. In instances where family members or children experience health issues, they tend to attribute them to their own bacterial carriage, leading to feelings of self-doubt, self-blame, and a detrimental cycle of self-deprecation and anxiety. Consequently, they become increasingly emotionally fragile and excessively fearful of occupational exposure.

“We are in an infectious disease unit where our patients are afflicted with serious contagious diseases. I am genuinely afraid of infecting my child, so I make a point to cleanse myself thoroughly twice at the hospital before having contact with my child at home. Sometimes, after being in close proximity to severely infected patients in the unit, I am terrified to go home. I constantly feel unclean” (N10).

“The department admitted a patient with AIDS, and it just so happens that I am the one providing care for her these days… I am really afraid that I am at risk of occupational exposure, and I feel like shaking every time I get close to her, but I am afraid of hurting the patient’s self-esteem, and what if I get infected with AIDS, my baby is only a month old!” (N14).

#### 3.3.3 Relative pressure

Most participants stated that, at times, their mothers-in-law would make special visits to the ward and seek them out during breaks. They insisted that they ate and drank certain foods or soups, citing their belief that it was their responsibility to consume these items for the sake of their children. The traditional Chinese culture maintains that these foods enhance breast milk production and provide essential nutrients. However, the reality is quite different, as these fatty foods not only fail to stimulate milk production, but also contribute to unhealthy weight gain and milk congestion. Sometimes, the daughters refused, but their mothers-in-law expressed a lack of understanding and even resorted to verbal mockery in front of their colleagues. Almost all participants emphasized that the most significant impact on their well-being, postpartum depression, and anxiety came from the violent language and behavior exhibited by family members.

“My mother-in-law firmly believes that consuming these soups will boost milk production, but this belief is misguided. She insisted that I drink soup, and if I declined, she would become upset. I gained 20 pounds, yet this had no effect on my milk production. These soups are far from healthy. I understand that she cares about me and I find it impossible to refuse her, but this kind of concern genuinely distresses me” (N11).

“I drew up recipes but they thought my recipes did not make sense and had to give me stewed pig’s trotters with soybeans, I really do not understand it, it’s all unscientific eating, I’ve been eating it for 3 days in a row now and I’ve really had enough!” (16).

### 3.4 Theme 4: coping strategies

#### 3.4.1 Self-adjustment

Some participants mentioned that they were not particularly bothered by verbal violence from patients as long as it did not involve severe harm. They considered such incidents to be common occurrences and not overly extreme. Moreover, they understand that some patients engage in aggressive behavior due to their illness (such as brain damage or mental disorders) and fear that reacting too strongly might startle them. By practicing self-comfort and regulating their emotions, they can alleviate some of their emotional burdens.

“My patient did not have malicious intent; he was going through a painful struggle. It was just a momentary outburst, and he genuinely apologized to me a week later” (N5).

“We often have patients in our department who accidentally injure us by mistake, and as long as it’s not a particularly serious injury, I usually do not keep it in mind, it’s the norm of the job, I’m used to it, they do not mean it” (N16).

#### 3.4.2 Effective communication

When faced with coldness or misunderstanding from leaders and colleagues, some participants believed that it was crucial to prevent conflict escalation. They emphasized the importance of expressing their difficulties, engaging in constructive communication, and seeking understanding and support. In situations where patients blame them, excessive explanations can be perceived as a way of evading responsibility and may lead to even more severe accusations. Therefore, these participants advocated adopting a perspective of empathy, providing appropriate explanations, and engaging in sincere and effective communication to effectively manage conflicts with patients.

“During this busy period in our department, I prepared gifts for day shift nurses as a token of appreciation to help me with my workload when I arrived late. I express my gratitude to them for their help with this study. When dealing with patient complaints, I requested that the nursing supervisor to join me in explaining my situation. The patient felt respected and no longer gave me a hard time” (N19).

“Sincerity is the most important thing in communication; although the patient did not understand why I showed up a little later in the day, I apologize sincerely and promise that I would definitely work more diligently and the patient was very understanding” (N6).

#### 3.4.3 Facing up to the dilemma

Some participants mentioned that they were not afraid of violence and could confidently devise suitable strategies for dealing with different situations. Mothers believe motherhood is the most important occupation worldwide. Having experienced childbirth and nurturing, they gained sufficient courage to confront any form of violence or adversity and triumph over them.

“Why should I be afraid? I am a mother and I strive to become a role model for my children. His mother was not weak and easily intimidated. As he grows up, he should also possess the courage to withstand pressure and violence” (N8).

“All things will be ok, the breastfeeding period will pass, the baby will grow up, my work will get better and better, things have to be resolved reasonably, you cannot worry too much, and you have to show the commitment of a mother and a qualified caregiver” (N10).

## 4 Discussion

Nurses are prone to physiological discomfort that affects the quality of their work during the breastfeeding period. Due to frequent breastfeeding at night and taking care of the baby, resulting in poor sleep quality, breastfeeding time often conflicting with work, and also experiencing some harassment incidents due to the physiological state of breastfeeding, all these adverse external influences will affect the nurses’ work and psychological state.

Breastfeeding is vital for infants as well as mothers and is an important way to build emotional connections and bonds that benefit the physical and mental health of both infants and mothers. During breastfeeding, mothers release hormones such as oxytocin and prolactin, which help to reduce stress and increase optimistic and positive feelings (30). However, breastfeeding poses a significant challenge for mothers. They often devote most of their time to caring for their babies, leading to extreme fatigue. Additionally, breastfeeding-related discomfort or breast lumps may occur (31). Some participants reported pain associated with breast engorgement. Although cancer has been ruled out as a possibility, it can still be attributed to milk stasis and mastitis. Milk stasis can be caused by various factors, and if left untreated over an extended period, it can result in mastitis, characterized by symptoms such as breast pain, hardness, swelling, flu-like symptoms, and in severe cases, the development of breast abscesses requires surgical intervention (32). Research indicates that workplace stress, unfavorable living environments, and increased life pressures increase the risk of mastitis. Working women during the breastfeeding period may be more prone to insufficient milk drainage, improper feeding techniques, delayed breastfeeding, shortened feeding durations, or use of formula milk, all of which can lead to blocked milk ducts and the onset of mastitis (33). Inadequate sleep and rest can negatively affect physical and mental well-being. Burns et al.’s cross-sectional study of breastfeeding mothers returning to work revealed that insufficient rest and demanding work schedules disrupted breastfeeding patterns, causing increased anxiety and a higher likelihood of depression (34). These findings are consistent with the results of our study. Furthermore, the participants encountered pressure and discrimination from their supervisors and colleagues, which adversely affected their physical and mental health. Additionally, partner involvement proved to be challenging. Some participants’ husbands had lower levels of education and limited knowledge about breastfeeding, resulting in a negative attitude towards breastfeeding, a lack of responsibility, and the perception that breastfeeding was solely the mother’s duty, thereby straining the relationship between the baby and father (35), further exacerbating conflicts, and hindering breastfeeding.

Some participants acknowledged that during the breastfeeding period, they experienced distinct physiological changes and encountered verbal and behavioral harassment from patients or their caregivers. Sexual harassment is a grave crime that poses a significant threat to social harmony. In China, the concept of “sexual harassment” was introduced relatively late from a legal perspective. Initially, the focus was primarily on raising awareness, without vigorously prosecuting harassers or providing effective tools for women to defend their rights (36). Consequently, legal and social mechanisms for addressing sexual harassment in China remain underdeveloped. However, incidents of sexual harassment are prevalent in modern society (37). In this study, most perpetrators engaged in such behaviors under the influence of alcohol. They deliberately evade legal and societal norms as well as moral education, exploiting their surroundings and social status to actively pursue malicious intentions (38). However, nurses who have experienced such harassment rarely report or accuse perpetrators. They believe that reporting might further complicate matters and expose them to higher risks than not reporting at all (37). Moreover, subsequent handling procedures following a report could increase time and cost during the breastfeeding period, consume energy, and potentially subject them to greater psychological pressure. In China’s male-dominated social and cultural environment, women who have been sexually harassed often face social stigma. They are frequently blamed, and their personal and family reputations may be tarnished (39). Consequently, when confronted with harassment, women carefully weigh the advantages and disadvantages and often choose to tolerate the situation as their ultimate outcome. A systematic review focusing on sexual harassment experienced by female nurses revealed its profound impact on their physical and mental well-being, emotions, social relationships, and broader social environment. Female nurses may experience anxiety, fear, disappointment, and helplessness (40), which aligns with our findings.

Many nurses get married and pregnant when they first enter the workforce, and then go through the process of giving birth and taking a leave of absence. When they return to work, their limited work experience makes them feel that their competence is out of touch with reality, and the pressure of their work increases sharply as they juggle their children and work, and their busy lives confuse them in their roles.

Currently, there is a significant gap between China’s healthcare service system and those of other developed countries. Additionally, the shortage of clinical nursing staff in China contributes to inadequate fine-tuned pre-employment training, resulting in lower levels of service skills and communication efficiency in some areas (17). Nurses under the age of 30 with limited experience often face a higher frequency of WPV because of a lack of job-matched expertise and insufficient nursing abilities. In this study, some patients and their families lacked knowledge and understanding of diseases and medical care process (41). Cognition plays a crucial role in judgment, reasoning, and decision-making, serving as an intuitive and reflective process that can enhance the accuracy of cognitive decisions. However, it can also give rise to predictable errors known as cognitive biases (42). When nurses lack effective communication skills, they may encounter communication barriers with patients, leading to cognitive bias in some patients. These biases can significantly influence true assessments and contribute to violent incidents.

In this study, breastfeeding nurses were subject to malicious targeting by some patients. One possible reason for this is that patients may perceive a loss of 1 hour of nursing care, leading to a sense of entitlement and potential disruption to their recovery process. As a result, they may express frustration with nursing staff. Being subjected to violence can severely impact the work confidence of nurses, causing a significant blow to their self-esteem. A longitudinal study suggested that self-esteem directly influences positive attitudes towards future work and indirectly affects self-efficacy levels (43). Low self-efficacy has a profound effect on job satisfaction, which in turn influences job performance, productivity, and work enthusiasm (44). This creates a detrimental cycle in which nurses experience role conflict, resulting in lower-quality nursing work, which aligns with the findings of our research.

Working in a hospital, the risk of occupational exposure is often faced, and this risk is magnified for nurses who have just returned to work after maternity leave, and who are nervous and anxious about breastfeeding, and who often add a lot of psychological burdens to themselves by worrying too much about occupational exposure, as well as the care from their husband’s mother, who can be a “burden of love” for them.

Infectious disease nurses face a high risk of occupational exposure. In this study, some participants displayed behaviors consistent with obsessive-compulsive disorders, including repetitive intrusive thoughts, intentions, and impulses. For instance, they engage in repeated hygiene rituals before interacting with their children at home. These compulsive behaviors may stem from a lack of postpartum security, strained family relationships, and a demanding work pace. These factors can lead to long-term concerns or excessive preoccupation with certain issues, resulting in a decline in reality perception and the outward manifestation of compulsive behaviors (45). A small subset of participants experienced internalized stigma, perceiving themselves as socially worthless and adopting negative stereotypes and characteristics such as impurities, contamination, and a sense of being unfit for contact with family members (46). Research suggests a positive correlation between the severity of obsessive-compulsive behavior and internalized stigma. Self-directed psychological and cognitive violence diminishes the quality of life and functioning of breastfeeding mothers (47).

In China, mothers-in-law play a crucial role during the breastfeeding period for infant care. However, the findings of this study revealed that the majority of participants expressed dissatisfaction with their mothers-in-law’s behavior. Participants described their mothers-in-law as self-centered, excessively confident, and heavily influenced by traditional Chinese culture, leading them to impose outdated breastfeeding practices and parenting guidelines. The strained relationship between mothers-in-law and daughters was identified as a long-standing family conflict and a significant cause of anxiety and depression among participants. Supporting this, a cross-sectional study conducted by Yu et al. in Hunan Province found that a problematic mother- and daughter-in-law relationship was a contributing factor to depression in breastfeeding women (48), which is consistent with the findings of our research. The tense relationship can have a detrimental effect on the emotional well-being of breastfeeding women, even when the mother-in-law’s actions may be well-intentioned, as they are often subconsciously rejected by the daughter-in-law.

The power of mothers was great. The change in their role brings them great self-soothing and self-healing abilities; they are more compassionate and understanding of others, and at the same time, they have the ability to protect themselves, and it is as if they have an extra layer of armor after becoming mothers.

In this study, some nurses employed self-healing methods to alleviate the pain caused by WPV. By engaging in healing practices and releasing their inner emotions, they enhanced their resilience when faced with distress and alleviated anxiety (49). These nurses actively chose to extend forgiveness towards patient accusations and verbal abuse even when feeling aggrieved or panicked. Their compassionate nature led them to empathize with the suffering experienced by patients, fostering an emotional connection and reducing their tendency to judge others’ behaviors. Furthermore, they develop greater tolerance of their own hardships when witnessing others in pain (50). Remarkably, almost all the participants expressed a strong commitment to their profession and had no intention of leaving. They recognized the importance of their jobs in providing for their children and motivating them to confront challenges with courage. By actively engaging in communication, conflict resolution, and reconciliation with themselves, colleagues, and patients, they enhanced their self-efficacy. This proactive approach was driven by their desire for personal growth and survival as they transformed their attitudes and bolstered their confidence in themselves and their work (44). Becoming mothers imbued them with tremendous courage and strength.

This study should be interpreted with caution because of several limitations. One potential limitation is the presence of recall and nonresponse biases. The interviewed nurses may have had memory bias, leading to inaccuracies in their recollection of events. Furthermore, some nurses may have chosen not to disclose their experiences in detail because of various concerns. These concerns include fear of facing pressure from superiors, apprehension about potential negative reactions from colleagues, and discomfort in discussing personal experiences openly. Consequently, the study may not have fully captured the breadth of experience and the impact of WPV among less experienced breastfeeding nurses.

## 5 Conclusion

This study delved deeply into the experiences of less experienced breastfeeding nurses who have encountered WPV. The findings underscore the significance of various factors in rebuilding their confidence; fostering healthy breastfeeding practices; alleviating feelings of inferiority, sensitivity, anxiety, and depression; and ensuring stable nursing service quality to promote their overall well-being. The key factors identified included the implementation of reasonable and supportive breastfeeding habits and environments; cultivating a friendly and harmonious work atmosphere; receiving understanding and care from family members, leaders, colleagues, and patients; awakening self-protective awareness; providing proactive and timely psychological counseling and treatment; offering training in nursing skills and communication techniques; ensuring support and protection from leaders during incidents of violence; rational distribution of work tasks; and attention to nurses’ mental health. Furthermore, hospitals should establish welfare policies, incentives, and comfort systems specifically tailored for breastfeeding women, while also nurturing nurses’ self-soothing abilities. Addressing and minimizing the harm caused by violence can potentially increase job satisfaction among nurses, foster the physical and mental well-being of breastfeeding nurses, and promote healthy breastfeeding habits. It is crucial to recognize the sensitivity of breastfeeding women’s emotions and handle their experiences with care, as mishandling can significantly impact the health of both mothers and children.

## Data availability statement

The raw data supporting the conclusions of this article will be made available by the authors, without undue reservation.

## Ethics statement

The studies involving humans were approved by Academic Ethics Committee of Binzhou Medical University. The studies were conducted in accordance with the local legislation and institutional requirements. The participants provided their written informed consent to participate in this study.

## Author contributions

RC: Conceptualization, Data curation, Formal analysis, Investigation, Software, Writing – original draft, Writing – review & editing. RW: ___. DW: Conceptualization, Data curation, Funding acquisition, Methodology, Project administration, Supervision, Validation, Visualization, Writing – original draft, Writing – review & editing. QW: Project administration, Resources, Supervision, Funding acquisition, Methodology, Software, Writing – review & editing. XL: Funding acquisition, Resources, Software, Visualization, Writing – review & editing.
